# Supplementary material for: Clinical criteria to exclude acute vascular pathology on CT angiogram in patients with dizziness
Source: PLoS One. 2023 Mar 9;18(3):e0280752. doi: 10.1371/journal.pone.0280752 (PMC9997874; doi:10.1371/journal.pone.0280752)
Supplement: S1 File — (DOCX) [file pone.0280752.s001.docx]

**S1 Supporting Information**

*Supporting Information for:*

*Clinical Criteria to Exclude Acute Vascular Pathology on CT Angiogram in Patients with Dizziness*

**Table of Contents**

- Item 1: Patient selection for CTA evaluation.
- Item 2: Established vascular risk factors
- Item 3: Definition of large vessel occlusion (LVO)
- Item 4: Additional details of feature encoding
- Item 5: Schematic of feature selection process for decision rule derivation
- Item 6: Categorization of 47 acute vascular findings in the training and validation cohorts
- Item 7: Details regarding the definition of current/former smoking and “long term” use of medications
- Item 8: Two-by-two table of patients excluded (vs. not excluded) by decision rule and those with (vs. without) acute vascular pathology in the derivation phase
- Item 9: Decision rule performance during the derivation phase, validation phase, and sensitivity analysis.
- Item 10: Comparison of decision rule to NIHSS performance

**Item 1: Patient selection for CTA evaluation.** In the three emergency departments included in this study, the use of CTA head and neck was based on clinical suspicion of stroke/LVO as determined by the evaluating clinician. We are not aware of any widely adopted/universal guidelines for the neuroimaging of dizzy patients; CTA use was expected to be based on clinical judgement.

With regard to contrast administration, patients without anaphylactic reaction to (iodinated) CT contrast and with eGFR>30 were eligible to receive CTA. Patients with moderate allergies to contrast (e.g., urticaria) were eligible after pre-medication. Patients in whom the suspicion for underlying vascular pathology was sufficiently high that the benefits were thought to outweigh the risks could receive CTA regardless of eGFR, on a case-by-case basis as determined by the evaluating clinician. In the “stroke code” scenarios used for the sensitivity analysis, patients are also eligible to receive CTA head and neck regardless of renal function, as benefits are expected to outweigh risks.

At the three emergency departments, CT/CTA imaging was more accessible than MRI, and utilized for initial evaluations. Expect this scenario to similar to most acute care settings. We recognize that in select institutions within the US or worldwide, emergency departments may have access to rapid evaluation by MRI, in which case MR diffusion imaging (with or without MRA) may offer alternative workflows. We expect the results of our work to remain applicable to the detection of acute vascular pathology in general, and therefore relevant sites with rapid MRI capability, in assisting selection for MRA head and neck evaluation.

| **Category** | **Clinical Characteristic** |
| --- | --- |
| Demographic information | Age, sex |
| Medical history risk factors | hypertension, diabetes, hyperlipidemia/dyslipidemia, overweight/obesity, prior stroke/transient ischemic attack, coronary artery disease, intracranial atherosclerosis, extracranial atherosclerosis, atrial fibrillation, other arrythmias, hypercoagulable states (including genetic, hyperhomocysteinemia, malignancy), congenital and structural heart diseases (including patent foramen ovale), cardiac tumors, obstructive sleep apnea, Moyamoya disease and other vasculopathies/vasculitis, history of dissection, genetic syndromes |
| Social, family, and substance use history | Family history of stroke, dietary patterns/ metabolic syndrome, physical inactivity, smoking history, drinking history, stimulant/illicit drug use |
| Associated signs and symptoms | headache, ataxia, imbalance, unilateral weakness, slurred speech, nausea, vomiting, double vision, nystagmus, Babinski’s sign, NIHSS |

**Item 2: Established vascular risk factors.** Known medical history and physical exam factors for LVO and stroke were used to inform the first phase of decision rule derivation.

**Item 3: Definition of large vessel occlusion (LVO).** We considered occlusion of any of the following to constitute LVO: internal carotid artery, middle cerebral artery M1 segment, anterior cerebral artery A1 segment, posterior cerebral artery P1 segment, vertebral artery, and basilar artery.

**Item 4: Additional details of feature encoding.** Any free-text abnormal findings were converted to categorical variables reflecting presence of documentation, with absent documentation represented by a null value. NIHSS and GCS were coded as two variables each – the first reflecting whether the assessment was recorded in the medical chart, the second representing the reported value if recorded, or a normal value if missing. Imputation and use of missing indicator variables may bias prediction models; the choice of single imputation by normal values was done to ensure that all potential bias would reduce specificity, while preserving high sensitivity, as would be required for a clinically useful decision rule. (Ultimately, no features requiring missing indicator variables were found to be sufficiently predictive to be included in the model.)

**Item 5: Schematic of feature selection process for decision rule derivation.** Features were added to a (decision) list based on feature importance and path location within decision trees. Established risk factors were added first, then other, potentially less well-recognized predictors were included.


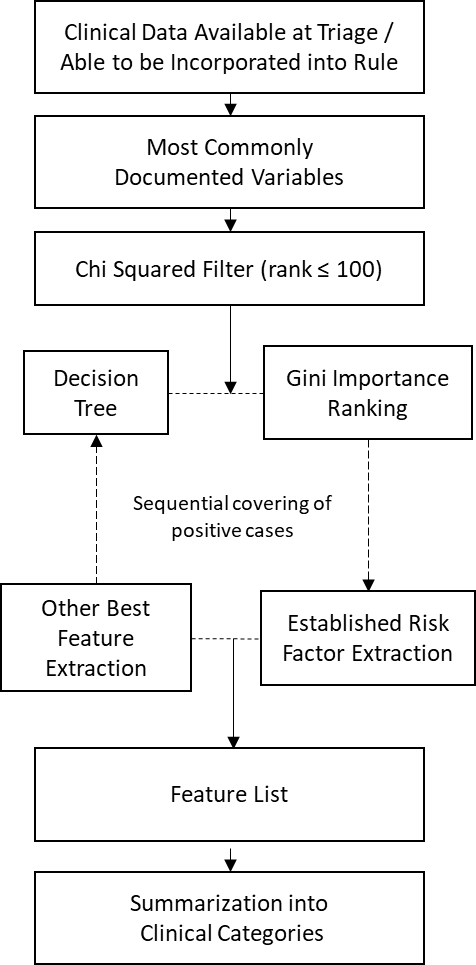


**Item 6: Categorization of 47 acute vascular findings in the training and validation cohorts.** There were 31 cases with large vessel occlusion and 16 other acute vascular abnormalities. These included 8 smaller arterial occlusions, 1 ruptured aneurysm, and 7 non-occlusive dissections. Most vascular abnormalities occurred in the posterior circulation of the brain, though two of the large vessel occlusions and 6 of the other acute vascular pathologies occurred in the anterior circulation.

| **31 large vessel occlusions** | **16 other acute vascular findings** |
| --- | --- |
| **26 posterior circulation**   - 18 single vertebral artery - 2 bilateral vertebral arteries - 3 basilar artery and posterior cerebral artery (P1) - 1 basilar artery only - 2 posterior cerebral artery (P1)   **3 anterior circulation**   - 1 common carotid artery and internal carotid artery - 2 internal carotid artery only   **2 anterior and posterior circulation**   - 2 internal carotid artery and vertebral artery | **8 smaller arterial occlusions**   - 2 posterior inferior cerebellar artery - 1 anterior inferior cerebellar artery - 2 posterior cerebral artery (P2-P3) - 1 anterior temporal artery - 2 middle cerebral artery (M2-M3) |
|  | **1 ruptured aneurysm**   - 1 anterior communicating artery |
|  | **7 non-occlusive dissections**   - 3 single vertebral artery - 1 bilateral vertebral artery - 2 internal carotid artery - 1 subclavian artery |

**Item 7: Details regarding the definition of current/former smoking and “long term” use of medications.** Features identified for the decision rule correspond to specific ICD-10 codes, coded at the discretion of clinical providers and nursing staff. While there are not strict criteria for application, smoking status may be approximated by a threshold of above 100 cigarettes over the lifetime (a figure used by the CDC), and whether patients are still smoking (current) or not (former).

There are no specific time or exposure criteria to designate long term use of aspirin, antithrombotics/antiplatelets, or anticoagulants according to ICD documentation. The designation of long-term use is based on medical indication, such as for the long-term treatment of a condition or for prophylactic use. Examples include for the prevention of deep vein thrombosis or as treatment of a chronic condition. These designations do not include administration for a brief period of time to treat an acute illness or injury. (For additional details, see links for relevant years at: https://www.cms.gov/medicare/coding/icd10, accessed: 11/20/2022.)

**Item 8: Two-by-two table of patients excluded (vs. not excluded) by decision rule and those with (vs. without) acute vascular pathology in the derivation phase.** The derivation phase cohort contained 41 patients with acute vasculature pathology (1031 without). The decision rule “excluded” acute pathology in 603 patients (acute pathology *not* excluded in 469). The 41 patients with acute pathology were all appropriately categorized as “NOT low risk.”

| ***Derivation phase cohort*** | ***Excluded* by decision rule (“low risk”)** | ***Not excluded* by decision rule (“NOT low risk”)** | **Total patients** |
| --- | --- | --- | --- |
| ***With* acute vascular pathology** | 0 | 41 | 41 |
| ***Without* acute vascular pathology** | 603 | 428 | 1031 |
| **Total patients** | 603 | 469 | 1072 |

**Item 9: Decision rule performance during the derivation phase, validation phase, and sensitivity analysis.** Performance characteristics are assessed in differing cohorts at each phase. 95% confidence intervals are listed in parentheses.

| **Test** | **Sensitivity** | **Specificity** | **Positive Predictive Value (PPV)** | **Negative Predictive Value (NPV)** | **Predicted Negative (PN)** |
| --- | --- | --- | --- | --- | --- |
| Decision Rule (derivation) | 1.00 (0.91-1.00) | 0.59 (0.56-0.62) | 0.09 (0.07-0.12) | 1.00 (0.99-1.00) | 0.56 (0.53-0.59) |
| Decision Rule (validation) | 1.00 (0.61-1.00) | 0.53 (0.48-0.58) | 0.04 (0.02-0.07) | 1.00 (0.98-1.00) | 0.52 (0.47-0.57) |
| Decision Rule (sensitivity analysis) | 1.00 (0.76-1.00) | 0.51 (0.39-0.62) | 0.26 (0.16-0.40) | 1.00 (0.90-1.00) | 0.43 (0.33-0.54) |

**Item 10: Comparison of Decision Rule to NIHSS performance.** NIHSS was uncommonly reported for patients presenting with dizziness, only 23.2% (331/1429) of all cases included in this study. In the validation cohort, NIHSS was reported in 18.8% of cases (67/357), including 83% (5/6) cases of acute vascular pathology and 100% (3/3) cases of LVO. Considering cases where a value was documented in the medical record, the commonly used NIHSS ≤ 7 to exclude vascular pathology had a sensitivity of 0% (0/5) and specificity of 89.6% (60/67). Using a minimal value of NIHSS = 0 to exclude vascular pathology had a sensitivity of 60% (3/5) and specificity of 44.8% (30/67). (Note: Because NIHSS was uncommonly documented, exact performance cannot be inferred from these data, and calculated values are provided as an approximation; however, imputing NIHSS = 0 for missing documentation would further decrease sensitivity for all NIHSS cut-offs.)
