# Supplementary material for: Clinical criteria to exclude acute vascular pathology on CT angiogram in patients with dizziness
Source: PLoS One. 2023 Mar 9;18(3):e0280752. doi: 10.1371/journal.pone.0280752 (PMC9997874; doi:10.1371/journal.pone.0280752)
Supplement: Attachment — Submitted filename: Reviewer Comments.docx [file pone.0280752.s002.docx]

**We would first like to thank the academic editor and reviewers for their thorough and insightful comments. We have addressed all suggestions, and are amenable to further revisions should they help clarify this work.**

ONE-D-22-23945

Clinical Criteria to Exclude Acute Vascular Pathology on CT Angiogram in Patients with Dizziness

PLOS ONE

Dear Dr. Tu,

Thank you for submitting your manuscript to PLOS ONE. After careful consideration, we feel that it has merit but does not fully meet PLOS ONE’s publication criteria as it currently stands. Therefore, we invite you to submit a revised version of the manuscript that addresses the points raised during the review process.

==============================
ACADEMIC EDITOR: All issues raised by expert reviewers are required. I recommend inclusion of criteria on how to judje application of *contrast* CTA head and neck exam (clinical values of biochemical data on renal function, etc.) to 3 institutes studied in supplementary data. I understand CTA is easy to test in ED in the US; however in some other countries (e.g., Germany, Japan), non-contrast MR diffusion images (<30sec) and MRA (~30 min) could be proceeded to detect LVO in the posterior circulation for EVT.

**Thank you so much for this thoughtful perspective. We have elaborated on the selection of patients for contrast CTA head and neck, including with regard to renal function. We very much agree that where available, non-contrast MR diffusion and/or MRA offers the potential for alternative workflows – we have also provided additional discussion on these scenarios. The additions are within S1 Supporting Information and referenced in *Methods (Setting and Design)*. The Supporting Information is re-numbered/reformatted throughout.**

 Please include the following items when submitting your revised manuscript:

- A rebuttal letter that responds to each point raised by the academic editor and reviewer(s). You should upload this letter as a separate file labeled 'Response to Reviewers'.
- A marked-up copy of your manuscript that highlights changes made to the original version. You should upload this as a separate file labeled 'Revised Manuscript with Track Changes'.
- An unmarked version of your revised paper without tracked changes. You should upload this as a separate file labeled 'Manuscript'.

If you would like to make changes to your financial disclosure, please include your updated statement in your cover letter. Guidelines for resubmitting your figure files are available below the reviewer comments at the end of this letter.

==============================

Please submit your revised manuscript by Dec 23 2022 11:59PM. If you will need more time than this to complete your revisions, please reply to this message or contact the journal office at [plosone@plos.org](mailto:plosone@plos.org). When you're ready to submit your revision, log on to https://www.editorialmanager.com/pone/ and select the 'Submissions Needing Revision' folder to locate your manuscript file.

If applicable, we recommend that you deposit your laboratory protocols in protocols.io to enhance the reproducibility of your results. Protocols.io assigns your protocol its own identifier (DOI) so that it can be cited independently in the future. For instructions see: [https://journals.plos.org/plosone/s/submission-guidelines#loc-laboratory-protocols](https://nam12.safelinks.protection.outlook.com/?url=https%3A%2F%2Fjournals.plos.org%2Fplosone%2Fs%2Fsubmission-guidelines%23loc-laboratory-protocols&data=05%7C01%7Clong.tu%40yale.edu%7C3b56fb61fe484f2c46fe08dac19e4140%7Cdd8cbebb21394df8b4114e3e87abeb5c%7C0%7C0%7C638035183216195709%7CUnknown%7CTWFpbGZsb3d8eyJWIjoiMC4wLjAwMDAiLCJQIjoiV2luMzIiLCJBTiI6Ik1haWwiLCJXVCI6Mn0%3D%7C3000%7C%7C%7C&sdata=s8ClZYDpvcOmT%2BAy6dKdWgqEgX2Al9MZvhj1IHRvqk0%3D&reserved=0). Additionally, PLOS ONE offers an option for publishing peer-reviewed Lab Protocol articles, which describe protocols hosted on protocols.io. Read more information on sharing protocols at [https://plos.org/protocols?utm_medium=editorial-email&utm_source=authorletters&utm_campaign=protocols](https://nam12.safelinks.protection.outlook.com/?url=https%3A%2F%2Fplos.org%2Fprotocols%3Futm_medium%3Deditorial-email%26utm_source%3Dauthorletters%26utm_campaign%3Dprotocols&data=05%7C01%7Clong.tu%40yale.edu%7C3b56fb61fe484f2c46fe08dac19e4140%7Cdd8cbebb21394df8b4114e3e87abeb5c%7C0%7C0%7C638035183216195709%7CUnknown%7CTWFpbGZsb3d8eyJWIjoiMC4wLjAwMDAiLCJQIjoiV2luMzIiLCJBTiI6Ik1haWwiLCJXVCI6Mn0%3D%7C3000%7C%7C%7C&sdata=1Y9AEC2AZmbigvoCRDFFtiuwoktsXi6mVqpZfhmOIPE%3D&reserved=0).

We look forward to receiving your revised manuscript.

Kind regards,

Tatsushi Mutoh
Academic Editor
PLOS ONE

Journal Requirements:

When submitting your revision, we need you to address these additional requirements.

1. Please ensure that your manuscript meets PLOS ONE's style requirements, including those for file naming. The PLOS ONE style templates can be found at

<https://journals.plos.org/plosone/s/file?id=wjVg/PLOSOne_formatting_sample_main_body.pdf> and

[https://journals.plos.org/plosone/s/file?id=ba62/PLOSOne_formatting_sample_title_authors_affiliations.pdf](https://nam12.safelinks.protection.outlook.com/?url=https%3A%2F%2Fjournals.plos.org%2Fplosone%2Fs%2Ffile%3Fid%3Dba62%2FPLOSOne_formatting_sample_title_authors_affiliations.pdf&data=05%7C01%7Clong.tu%40yale.edu%7C3b56fb61fe484f2c46fe08dac19e4140%7Cdd8cbebb21394df8b4114e3e87abeb5c%7C0%7C0%7C638035183216195709%7CUnknown%7CTWFpbGZsb3d8eyJWIjoiMC4wLjAwMDAiLCJQIjoiV2luMzIiLCJBTiI6Ik1haWwiLCJXVCI6Mn0%3D%7C3000%7C%7C%7C&sdata=Eteo72ol0nznfRm7msb4Og8PH%2FnU%2FtbYVQ3M3mODgtw%3D&reserved=0)

**Done. Thank you.**

2. Thank you for stating the following in the Competing Interests section:

“I have read the journal's policy and the authors of this manuscript have the following competing interests: LT reported receiving royalties for 2 textbooks, Search Pattern: A Systematic Approach to Diagnostic Imaging (2020) and A Brief Guide to the Neuroradiology Fellowship (2021), and was a student in the Yale University Investigative Medicine Program, which receives funding from the National Center for Advancing Translational Science, a component of the National Institutes of Health (NIH). KS reported receiving grants from Hyperfine during the conduct of the study and from the NIH, American Heart Association, and Biogen outside the submitted work. KS also reported receiving personal fees from Zoll (data and safety monitoring board chair), Alva Equity, and Cerovasc outside the submitted work. AV reported receiving grants from the US Centers for Medicare and Medicaid Services, Moore Foundation, American College of Radiology, and American College of Emergency Physicians outside the submitted work. No other disclosures were reported. The contents of this work are solely the responsibility of the authors and do not necessarily represent the official view of the NIH.”

Please confirm that this does not alter your adherence to all PLOS ONE policies on sharing data and materials, by including the following statement: "This does not alter our adherence to PLOS ONE policies on sharing data and materials.” (as detailed online in our guide for authors <http://journals.plos.org/plosone/s/competing-interests>).  If there are restrictions on sharing of data and/or materials, please state these. Please note that we cannot proceed with consideration of your article until this information has been declared.

Please include your updated Competing Interests statement in your cover letter; we will change the online submission form on your behalf.

**We have added the relevant statement. Updated Competing Interests are included in the cover letter.**

3.  Please include your full ethics statement in the ‘Methods’ section of your manuscript file. In your statement, please include the full name of the IRB or ethics committee who approved or waived your study, as well as whether or not you obtained informed written or verbal consent. If consent was waived for your study, please include this information in your statement as well.

**Done. Thank you.**

4. We note that you have stated that you will provide repository information for your data at acceptance. Should your manuscript be accepted for publication, we will hold it until you provide the relevant accession numbers or DOIs necessary to access your data. If you wish to make changes to your Data Availability statement, please describe these changes in your cover letter and we will update your Data Availability statement to reflect the information you provide.

**Thank you, yes. We will be providing repository information with acceptance.**

5. Please include captions for your Supporting Information files at the end of your manuscript, and update any in-text citations to match accordingly. Please see our Supporting Information guidelines for more information: [http://journals.plos.org/plosone/s/supporting-information](https://nam12.safelinks.protection.outlook.com/?url=http%3A%2F%2Fjournals.plos.org%2Fplosone%2Fs%2Fsupporting-information&data=05%7C01%7Clong.tu%40yale.edu%7C3b56fb61fe484f2c46fe08dac19e4140%7Cdd8cbebb21394df8b4114e3e87abeb5c%7C0%7C0%7C638035183216195709%7CUnknown%7CTWFpbGZsb3d8eyJWIjoiMC4wLjAwMDAiLCJQIjoiV2luMzIiLCJBTiI6Ik1haWwiLCJXVCI6Mn0%3D%7C3000%7C%7C%7C&sdata=vmxKVp%2BfFG9LBRtfgkBNV85WIcJAGsONvEM0PSIz3Uk%3D&reserved=0).

**We have done this. There is only one supporting information file, containing 10 items. Please feel free to edit/remove the details provided.**

6. Please review your reference list to ensure that it is complete and correct. If you have cited papers that have been retracted, please include the rationale for doing so in the manuscript text, or remove these references and replace them with relevant current references. Any changes to the reference list should be mentioned in the rebuttal letter that accompanies your revised manuscript. If you need to cite a retracted article, indicate the article’s retracted status in the References list and also include a citation and full reference for the retraction notice.
**Done. Thank you.**

[Note: HTML markup is below. Please do not edit.]

Reviewers' comments:

Reviewer's Responses to Questions

**Comments to the Author**

1. Is the manuscript technically sound, and do the data support the conclusions?

The manuscript must describe a technically sound piece of scientific research with data that supports the conclusions. Experiments must have been conducted rigorously, with appropriate controls, replication, and sample sizes. The conclusions must be drawn appropriately based on the data presented.

Reviewer #1: Yes

Reviewer #2: Yes

2. Has the statistical analysis been performed appropriately and rigorously?

Reviewer #1: Yes

Reviewer #2: I Don't Know

3. Have the authors made all data underlying the findings in their manuscript fully available?

The [PLOS Data policy](https://nam12.safelinks.protection.outlook.com/?url=http%3A%2F%2Fwww.plosone.org%2Fstatic%2Fpolicies.action%23sharing&data=05%7C01%7Clong.tu%40yale.edu%7C3b56fb61fe484f2c46fe08dac19e4140%7Cdd8cbebb21394df8b4114e3e87abeb5c%7C0%7C0%7C638035183216195709%7CUnknown%7CTWFpbGZsb3d8eyJWIjoiMC4wLjAwMDAiLCJQIjoiV2luMzIiLCJBTiI6Ik1haWwiLCJXVCI6Mn0%3D%7C3000%7C%7C%7C&sdata=IECvCvxRoWY4tyzAmwB%2Bb9wt0Vkjc1KzXN8MaqGXJfA%3D&reserved=0) requires authors to make all data underlying the findings described in their manuscript fully available without restriction, with rare exception (please refer to the Data Availability Statement in the manuscript PDF file). The data should be provided as part of the manuscript or its supporting information, or deposited to a public repository. For example, in addition to summary statistics, the data points behind means, medians and variance measures should be available. If there are restrictions on publicly sharing data—e.g. participant privacy or use of data from a third party—those must be specified.

Reviewer #1: No

Reviewer #2: Yes

4. Is the manuscript presented in an intelligible fashion and written in standard English?

PLOS ONE does not copyedit accepted manuscripts, so the language in submitted articles must be clear, correct, and unambiguous. Any typographical or grammatical errors should be corrected at revision, so please note any specific errors here.

Reviewer #1: Yes

Reviewer #2: Yes

5. Review Comments to the Author

Please use the space provided to explain your answers to the questions above. You may also include additional comments for the author, including concerns about dual publication, research ethics, or publication ethics. (Please upload your review as an attachment if it exceeds 20,000 characters)

Reviewer #1: The paper entilted "Clinical Criteria to Exclude Acute Vascular Pathology on CT Angiogram in Patients with
Dizziness" is well written. I have no concern about the methods, results, and data interpretations. There is only one minor issue need the authors to revise:
“Review of system” was incorrectly abbreviated as “PE” in the paragraph of data collection.

**Thank you so much for the consideration. Yes, this was an oversight. We have made the correction.**

Reviewer #2: The purpose of this retrospective cross-sectional study was to identify a subpopulation of dizzy patients with near zero probability of acute vascular pathology detectable on CTA. The authors created a decision rule consisting of clinical factors that could exclude all cases of acute vascular pathology on CTA. The proposed sensitivity of the decision rule (100%) appears to be better than that of any thresholds of NIHSS scores up to 8 points.
This study documents interesting features concerning that as many as half of currently conducted head and neck CTA was avoidable.
I agree with the authors that this decision rule which was made to maximize sensitivity can potentially be a useful rule-out tool to detect dizzy patients who don’t need to undergo CTA, with future research assessment.
However, I have some suggestions for clarifying some aspects of the manuscript.

Specific points to be addressed are as follows.

1) Abstract (conclusion):
It is questionable to conclude that, "A collection of clinical factors may be able to “exclude” acute vascular pathology in up to half of the patients presenting to the ED with dizziness" since this study has excluded dizzy patients not receiving CTA head and neck, and targets acute vascular pathology detected on CTA. I recommend that the authors should tone down the statement as written in the discussion/conclusion of the manuscript and clarify that "acute vascular pathology” means that detectable on CTA.

**Yes. We agree completely. The original statement was inexact. We have updated the relevant line in the abstract and clarified a similar line in the discussion/conclusion.**

2) Methods:
(3rd Para) Data Collection
(2nd page of the methods, 1st line) The authors wrote that “review of systems (PE)”, but isn’t it ROS?

**Yes. This was an error. Thank you. We have made the correction.**

3) Results:
(3rd Para) Decision Rule Performance
(4th page of the results, 5th line) The authors described the sensitivity and specificity of the decision rules applied in the derivation phase cohort. Since the rule was composed of negative forms and made as exclusion criteria, sensitivity and specificity seem complicated at first sight. Adding the conventional two-by-two table of patients with/without acute vascular pathology on CTA and patients included in the rules (low risk)/NOT included in the rules (NOT low risk), as Supplemental data would be helpful for readers.

**This is a great point. We have added such a table to S1 Supporting Information and referenced it in-text in the results.**

4)Table1, Table2:
There are some criteria with “long-term”, such as long-term smoking, and long-term medication use. How long do you define the length of “long-term”? Please describe.

**Thank you. We have described these designations further in S1 Supporting Information, with reference within the legends for Table 1 and Table 2. We would also like to note here that we noticed a typo in table 2 in enumerating *22* features summarized into 4 broad clinical categories. There are *23* features (two related to insulin use were previously listed on the same line) – we have made appropriate corrections in the legend/text. Please let us know if this is alright.**

5)Table 3:
The table compares the sensitivity and specificity of NIHSS and the clinical decision rule when applied to the validation cohort. However, the performance of the decision rule during the derivation phase and sensitivity analysis are also added. I think that those are not appropriate to add in parallel in this table since they are applied in the different cohorts. I would recommend the values of NIHSS and validation cohort and those of derived phase and sensitivity analysis are presented separately.

**Yes. We agree completely. We have removed the derivation and sensitivity analysis from this table. We have instead presented decision rule performance in the differing phases of the study in a table within the S1 Supporting Information, indicating that the measures are produced using differing patient cohorts. Thank you!**

6. PLOS authors have the option to publish the peer review history of their article ([what does this mean?](https://nam12.safelinks.protection.outlook.com/?url=https%3A%2F%2Fjournals.plos.org%2Fplosone%2Fs%2Feditorial-and-peer-review-process%23loc-peer-review-history&data=05%7C01%7Clong.tu%40yale.edu%7C3b56fb61fe484f2c46fe08dac19e4140%7Cdd8cbebb21394df8b4114e3e87abeb5c%7C0%7C0%7C638035183216195709%7CUnknown%7CTWFpbGZsb3d8eyJWIjoiMC4wLjAwMDAiLCJQIjoiV2luMzIiLCJBTiI6Ik1haWwiLCJXVCI6Mn0%3D%7C3000%7C%7C%7C&sdata=fg%2F%2Fu3cefDDNoaJYLU%2BMHwizfWdFtXpvR9asBQ%2B4fX0%3D&reserved=0)). If published, this will include your full peer review and any attached files.


If you choose “no”, your identity will remain anonymous but your review may still be made public.


**Do you want your identity to be public for this peer review?** For information about this choice, including consent withdrawal, please see our [Privacy Policy](https://nam12.safelinks.protection.outlook.com/?url=https%3A%2F%2Fwww.plos.org%2Fprivacy-policy&data=05%7C01%7Clong.tu%40yale.edu%7C3b56fb61fe484f2c46fe08dac19e4140%7Cdd8cbebb21394df8b4114e3e87abeb5c%7C0%7C0%7C638035183216195709%7CUnknown%7CTWFpbGZsb3d8eyJWIjoiMC4wLjAwMDAiLCJQIjoiV2luMzIiLCJBTiI6Ik1haWwiLCJXVCI6Mn0%3D%7C3000%7C%7C%7C&sdata=mN6%2FLd5pEQvKaSd9q%2FSegf39dkRe9r8d6X6Zeynmdck%3D&reserved=0).

Reviewer #1: No

Reviewer #2: No

[NOTE: If reviewer comments were submitted as an attachment file, they will be attached to this email and accessible via the submission site. Please log into your account, locate the manuscript record, and check for the action link "View Attachments". If this link does not appear, there are no attachment files.]

While revising your submission, please upload your figure files to the Preflight Analysis and Conversion Engine (PACE) digital diagnostic tool, [https://pacev2.apexcovantage.com/](https://nam12.safelinks.protection.outlook.com/?url=https%3A%2F%2Fpacev2.apexcovantage.com%2F&data=05%7C01%7Clong.tu%40yale.edu%7C3b56fb61fe484f2c46fe08dac19e4140%7Cdd8cbebb21394df8b4114e3e87abeb5c%7C0%7C0%7C638035183216195709%7CUnknown%7CTWFpbGZsb3d8eyJWIjoiMC4wLjAwMDAiLCJQIjoiV2luMzIiLCJBTiI6Ik1haWwiLCJXVCI6Mn0%3D%7C3000%7C%7C%7C&sdata=MxYCc3lbtyxvvewxDdCPl9j5mxQ6%2BtkFghP5Mg2sgRE%3D&reserved=0). PACE helps ensure that figures meet PLOS requirements. To use PACE, you must first register as a user. Registration is free. Then, login and navigate to the UPLOAD tab, where you will find detailed instructions on how to use the tool. If you encounter any issues or have any questions when using PACE, please email PLOS at [figures@plos.org](mailto:figures@plos.org). Please note that Supporting Information files do not need this step.

**Done.**

*In compliance with data protection regulations, you may request that we remove your personal registration details at any time.* [*(Remove my information/details)*](https://nam12.safelinks.protection.outlook.com/?url=https%3A%2F%2Fwww.editorialmanager.com%2Fpone%2Flogin.asp%3Fa%3Dr&data=05%7C01%7Clong.tu%40yale.edu%7C3b56fb61fe484f2c46fe08dac19e4140%7Cdd8cbebb21394df8b4114e3e87abeb5c%7C0%7C0%7C638035183216195709%7CUnknown%7CTWFpbGZsb3d8eyJWIjoiMC4wLjAwMDAiLCJQIjoiV2luMzIiLCJBTiI6Ik1haWwiLCJXVCI6Mn0%3D%7C3000%7C%7C%7C&sdata=J6K5iVEe5%2BT9vD94RnoOBs8CBpqBTct0ZOKXR%2FmnI0Y%3D&reserved=0)*. Please contact the publication office if you have any questions.*
